# Pathogenic variants in chromatin‐related genes: Linking immune dysregulation to neuroregression and acute neuropsychiatric disorders

**DOI:** 10.1111/dmcn.16276

**Published:** 2025-02-22

**Authors:** Russell C. Dale, Shekeeb Mohammad, Velda X. Han, Hiroya Nishida, Himanshu Goel, Stuart G. Tangye, Georgina Hollway, Esther Tantsis, Deepak Gill, Shrujna Patel, Chromatin Immune‐Brain Group, Michael Lonergan, Melanie Wong, Kavitha Kothur, Erica Tsang

**Affiliations:** ^1^ Kids Neuroscience Centre, The Children's Hospital at Westmead, Faculty of Medicine and Health University of Sydney Sydney NSW Australia; ^2^ The Children's Hospital at Westmead Clinical School, Faculty of Medicine and Health University of Sydney Sydney NSW Australia; ^3^ Khoo Teck Puat‐National University Children's Medical Institute National University Health System Singapore Singapore; ^4^ Hunter Genetics Waratah NSW Australia; ^5^ School of Medicine and Public Health University of Newcastle Callaghan NSW Australia; ^6^ Garvan Institute of Medical Research Darlinghurst NSW Australia; ^7^ School of Clinical Medicine, Faculty of Medicine and Health UNSW Sydney Darlinghurst NSW Australia; ^8^ CIRCA (Clinical Immunogenomics Research Consortium Australia) Sydney NSW Australia

## Abstract

We report eight children with de novo pathogenic DNA variants in chromatin‐related genes: *MORC2, CHD7, KANSL1, KMT2D, ZMYND11, HIST1HIE, EP300,* and *KMT2B*. All children experienced infection or vaccine‐provoked neuroregression or abrupt‐onset neuropsychiatric syndromes. Most had delayed development (*n* = 6) before the first regression, and four had immune deficiency or autoimmunity (*n* = 4). At a mean age of 4 years 2 months (range 1–8 years), symptoms included infection‐provoked autistic/language regression (*n* = 6), cognitive decline (*n* = 3), gait deterioration (*n* = 3), or abrupt‐onset anxiety, obsessive‐compulsive disorder, and/or tics (*n* = 5). Three children had ongoing infection‐provoked deteriorations. Six children benefited from intravenous immunoglobulin (*n* = 3) or antibiotics (*n* = 4). Ribonucleic acid expression of the eight chromatin genes was similar in neuronal, glial, and peripheral leukocytes, unlike non‐chromatin neurodevelopmental genes, which have predominantly neuronal expression. These cases demonstrate the role of chromatin dysregulation in autistic regression and abrupt‐onset neuropsychiatric syndromes, potentially related to brain and immune gene dysregulation.

## Graphical abstract

Chromatin machinery influences how DNA is ‘wrapped’ around histones in the nucleosome of immune and brain cells.

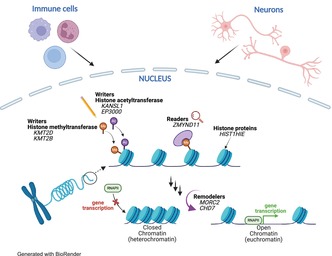

Plain language summary: https://onlinelibrary.wiley.com/doi/10.1111/dmcn.16299

**This original article is commented by Thompson on pages** 975–976 **of this issue.**

**Abbreviations**
OCD: obsessive‐compulsive disorder
PANS: paediatric acute‐onset neuropsychiatric syndrome
RNA: ribonucleic acid

**What this paper adds**

- DNA variation in chromatin‐related genes can cause immune dysregulation and infection‐provoked neuroregression and abrupt‐onset neuropsychiatric disorders.
- These observations link chromatin and immune/brain dysregulation, providing insights into poorly understood disorders such as autistic regression and PANS.

Although every cell in the body contains the same DNA sequence, the expression of genes—through the processes of transcription into ribonucleic acid (RNA) and subsequent translation into proteins—differs significantly among cell types. This differential gene expression enables cells to perform specialized functions required for various tissues and organs, and allows them to respond dynamically to environmental stimuli.^1^, ^2^ These adaptive cellular responses are largely mediated by changes in gene regulation through epigenetic mechanisms. Epigenetic machinery includes DNA modifications (e.g. methylation), chromatin structure, RNA modifications, short non‐coding RNAs, and posttranslational (protein) modifications.^1^ Chromatin, the condensed form of DNA and histone proteins, plays a critical role in this regulation. DNA is wrapped around histones to form nucleosomes and chemical modifications to histones (e.g. methylation) influence the chromatin state (Figure 1).^3^

**FIGURE 1:**
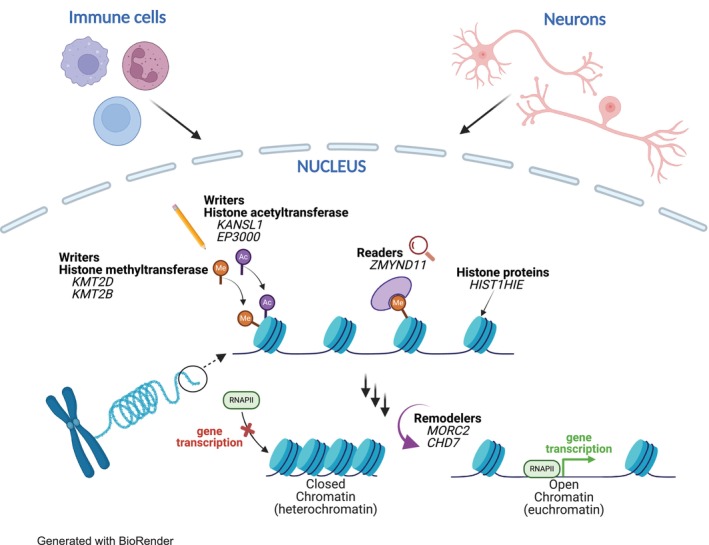
Immune and neuronal cell types contain chromatin machinery in the cell nucleus. Chromatin machinery influences how DNA is ‘wrapped’ around histones in the nucleosome: modifications such as writers, erasers, readers, and remodellers influence whether chromatin is closed (heterochromatin) and therefore less available for gene transcription mediated by polymerases such as RNAPII, or open (euchromatin) and therefore more available for gene transcription. Variations in chromatin‐related genes can affect gene transcription and downstream cellular function of both immune and neuronal cells, which may render them more vulnerable to environmental triggers such as infection.

Variations in genes that code for important chromatin‐associated proteins cause multiorgan genetic conditions.^4^, ^5^, ^6^ Some of these chromatin genes are enzymes involved in histone methylation such as methyltransferases and demethylases.^4^ We present a series of children with DNA variants in chromatin‐related genes who suffered infection‐provoked episodes of neuroregression and abrupt‐onset neuropsychiatric disorders, highlighting the potential role of chromatin dysregulation in immune‐brain interactions.

## METHOD

The cases were referred to the Children's Hospital at Westmead with clinical concerns of a possible neuroimmune process. The genetic investigations for all eight cases were performed in routine laboratories (after research testing in *n* = 2). The clinical data were captured from hospital records and corroborated with the families for accuracy. Case 4 has previously been published.^6^ All families have provided written informed consent for the publications of this case series. Using proteinatlas.org, we compared gene expression in different cell types using single‐cell RNA sequencing normalized transcripts per million data for neurons (excitatory and inhibitory), glia (astrocytes, oligodendrocytes, microglia), and peripheral leukocytes (T cell, B cell, granulocytes, monocytes).

## RESULTS

Eight children (six male and two female) were identified with de novo pathogenic or likely pathogenic variants in chromatin‐related genes (Table 1). Four of the genes encoded enzymes involved in histone acetylation or methylation (*KANSL1, KMT2D, EP300, KMT2B*), and the other genes (*MORC2, CHD7, ZMYND11, HIST1H1E*) were structural histone or chromatin binding/modelling genes (Table 1 and Figure 1). Although the DNA variants were found to be de novo (trio segregation), four cases had a first‐degree family history of a neurodevelopmental disorder, and four patients had a parent with an autoimmune/inflammatory disorder (Table 2).

**TABLE 1:** Genetic findings in eight children with DNA variation in chromatin‐related genes.

Case	Gene/microdel	Segregation	HGVS variant/ DECIPHER	Predicted ACMG pathogenicity (Franklin)	Gene function	Known associations (OMIM)
1	*MORC2*	De novo	MORC2:c.1321C > T Chr22‐31333850 G > A p.Arg441* NM_001303256.3	Likely pathogenic^a^	ATPase, Chromatin reader, DNA repair, transcriptional regulator	CMT disease axonal type 2z: Developmental delay, Impaired Growth, Dysmorphic Facies, and Axonal Neuropathy (DIGFAN)
2	*CHD7*	De novo	CHD7:c.7441C > T Chr8‐61769280 C > T p.Gln2481* NM_017780.4	Pathogenic	Chromatin remodeller, transcriptional regulator, ribosomal RNA biogenesis regulator	CHARGE syndrome
3	17q23.31 microdel^b^	De novo	17:45627800–46217040, 17q21.31 recurrent microdeletion syndrome	Pathogenic	KANSL1: histone (lysine) acetyltransferase complex subunit, chromatin writer (Histone H4 acetylation)	Koolen de Vries syndrome
4	*KMT2D*	De novo	KMT2D:c.12133C > T Chr12‐49426355 G > A p.Gln4045* NM_003482.4	Likely pathogenic	Histone (lysine) methyltransferase, chromatin writer, colocalizes with transcription factors	Kabuki syndrome
5	*ZMYND11*	De novo	ZMYND11:c.744_745del Chr10‐286041 CAT>C p.Cys249Serfs*2 NM_001370100.5	Pathogenic	Transcription repressor, chromatin reader, regulates RNA polymerase II elongation, binds histone H3.3 trimethylated at Lys‐36	*ZMYND11*‐related syndromic intellectual disability
6	*HIST1H1E*	De novo	HIST1H1E:c.253_254del Chr6‐26156870 CAA > C p.Lys85Glufs*34 NM_005321.3	Likely pathogenic	The linker histone, H1, interacts with DNA in nucleosomes and compacts chromatin	Rahman syndrome
7	*EP300*	De novo	EP300:c.4469C > A Chr22‐41568519 C > A p.Ala1490Asp NM_001429.4	Likely pathogenic	Histone acetyltransferase, chromatin writer	Rubinstein‐Taybi syndrome 2 Menke‐Hennekam syndrome 2
8	*KMT2B*	De novo	KMT2B: c.7288_7292dup Chr19‐36227718 G > GAGCTT p.Glu2432Alafs*9 NM_014727.3	Likely pathogenic	Histone (lysine) methyltransferase, chromatin eraser, colocalizes with transcription factors	Dystonia 28, childhood‐onset Intellectual developmental disorder, autosomal dominant 68

Abbreviations: ACMG, American College of American Genetics; CMT, Charcot–Marie–Tooth; DECIPHER, DatabasE of genomiC varIation and Phenotype in Humans using Ensembl Resources; HGVS, Human Genome Variation Society; OMIM, Online Mendelian Inheritance in Man; RNA, ribonucleic acid.

^a^ Most published variants in MORC2 are missense variants in the ATPase domain. The variant in case 1 is a nonsense variant resulting in a premature stop codon and predicted nonsense mediated decay. As MORC2 is haploinsufficient (pHaplo = 0.99) and the gene is under loss of function constraint (pLI = 1), the variant is absent in gnomAD v4, and has phenotypic fit with reported cases, the variant is categorized likely pathogenic pS2_supporting (ACMG).

^b^ It is generally accepted that the gene *KANSL1* is the pathogenic gene in this common microdeletion.

**TABLE 2:** Family history, clinical course, therapeutics, and ongoing diagnostics in eight children with DNA variants in chromatin‐related genes.

Case	Family history	Preceding development	Infection that provoked setback(s)	Neuroregression	Clinical course	Therapeutics	Current diagnostics and comorbidity
1 (female)	Schizophrenia (paternal) Natural killer infertility (maternal)	Typically developing to 4 years 6 months	4 years 6 months: over 1 month; gastroenteritis (*n* = 2), tonsilitis (*n* = 1), URTI (*n* = 1)	URTI merged into headache, separation anxiety, enuresis, confusion, loss of eye contact, inattention, aggression, hallucinations, OCD, weight loss, seizures, cognitive decline, tics	Developed neurodevelopmental syndrome, superimposed with ongoing infection‐provoked episodes of emotional behaviour, cognitive decline, and hallucinations (lasting weeks/months)	Lamotrigine, topiramate, quetiapine, guanfacine IVIG, augmentin,	10 years 6 months: ASD, intellectual disability (mild), anxiety, ODD, ADHD, OCD, tics. Precocious puberty
2 (female)	Alopecia (paternal) ASD (sister)	Global developmental delay, non‐verbal	1–5 years: recurrent respiratory, ear, and urine infections	From 5 years: tics, compulsive SIB with warfarin‐associated facial haematomas	Ongoing neurodevelopmental syndrome. Azithromycin resulted in cessation of dangerous SIB	Warfarin, clonidine Azithromycin	13 years: ASD, OCD, tics, intellectual disability (moderate) Choanal atresia, colobomas, SNHL, CHD, recurrent infections, spinal/hip dysplasia
3 (male)	ADHD (brother)	Global developmental delay, mild–moderate ID, hypotonia	6 years 6 months: URTI with croup	Day after croup, rapid onset social withdrawal, OCD, repetitive and rigid behaviours, separation anxiety, sensory issues, inattention	Continued severe emotional dysregulation in context of neurodevelopmental syndrome. Partial benefit with erythromycin	Quetiapine, aripiprazole, levetiracetam, thyroxine, GH Erythromycin	16 years: ASD, OCD, epilepsy, anxiety, intellectual disability (moderate) IgA deficiency, growth hormone deficiency, tethered spinal cord, neurogenic bladder, Hashimoto thyroiditis, eosinophilic oesophagitis
4 (male)	Nil	Visuo‐spatial processing	2 years 5 months: ear infection	Recurrent infection‐provoked 1–2 month episodes of cognitive decline, inattention, confusion, followed by partial recovery	Between 3–12 years, multiple episodes of infection‐provoked neuroregression episodes (1–2 months duration). Events ceased with ketogenic diet allowed cognitive progress	Methylphenidate, GH Ketogenic diet	15 years: CHD, growth hormone deficiency, coloboma, immune deficiency, recurrent ear infections, ADHD
5 (male)	Antiphospholipid syndrome (maternal)	Mild delay in speech and social development	5 years: postinfectious encephalitis	Steady decline into relapsing neuropsychiatric syndrome with cognitive decline, epilepsy, narcolepsy, tics, autism, emotional dysregulation, gait disorder	Steady decline over 10 years, but modified by three weekly intravenous immunoglobulin and rituximab	Fluoxetine, clonidine, lamotrigine, valproate, clobazam, modafinil, paliperidone IVIG, rituximab	18 years: ASD, intellectual disability (moderate), Tourette syndrome, emotional dysregulation, epilepsy, narcolepsy
6 (male)	ASD (brother) PTSD (paternal) Asthma (maternal)	Typically developing to 12 months	12 months: MMR vaccination	Day after vaccination, developed high fever, stopped speaking (lost 25 words), lost eye contact, repetitive stereotypical movements	Subsequent history of recurrent infection‐provoked behavioural change. 11 years: mycoplasma pneumonia induced OCD and severe anxiety	Fluoxetine Insulin Cannabis Azithromycin Tildrakizumab (IL‐23 blockade)	17 years: ASD, intellectual disability (severe), pica, anxiety, OCD, tics Recurrent infection, hidradenitis suppurativa, allergies, chronic diarrhoea Type 1 diabetes
7 (male)	JAK2 somatic variant myelofibrosis and thyroid cancer (maternal)	Speech delay, hyperactivity, inattention	7 years: URTI	Days after URTI, behaviour change and inability to walk (apraxia), stopped speaking, irritability, clingy, unresponsive	Improved after steroid and IVIg Ongoing neurocognitive syndrome	Dexamethasone IVIG	8 years: speech delay Hyperreflexia, anxiety, intellectual disability (mild)
8 (male)	Hyperemesis, iron deficient in pregnancy (maternal)	IUGR—poor growth, mild motor delay	2 years: URTI	Days after URTI, global regression, starting with speech, then motor and social (evolved over 1–2 months)	Stabilized but ongoing developmental syndrome	Clonidine	6 years: global developmental delay, hyperactivity

Abbreviations: ADHD, attention‐deficit/hyperactivity disorder; ASD, autism spectrum disorder; CHD, congenital heart disease; GH, Growth hormone; IUGR, in utero growth restriction; IVIG, intravenous immunoglobulin; MMR, measles, mumps, and rubella; OCD, obsessive‐compulsive disorder; ODD, oppositional defiant disorder; PTSD, posttraumatic stress disorder; SIB, self‐injurious behaviour; SNHL, sensorineural hearing loss; URTI, upper respiratory tract infection.

Six of eight children had existing developmental concerns before the first episode of neuroregression (Table 2). The first regression event occurred at a mean of 4 years 2 months of age (range 1–8 years). Seven of these events were triggered by infection, while one was linked to a vaccine (Table 2). In six patients, there was a rapid deterioration over the days after infection, whereas in two patients the neuroregression was more insidious over months and years. One patient had a well‐documented episode of encephalitis before an insidious regression over a decade (case 5). The dominant features of the incipient regression event were: autism/social/speech regression (*n* = 6), emotional dysregulation or anxiety (*n* = 5), obsessive‐compulsive disorder (OCD; *n* = 4), tics (*n* = 3), cognitive decline (*n* = 3), gait deterioration (*n* = 3), inattention (*n* = 2), incontinence (*n* = 2), seizures (*n* = 2), repetitive behaviour (*n* = 2), confusion (*n* = 2), self‐injurious behaviour (*n* = 2), aggression, hallucinations, weight loss, narcolepsy, sensory issues (*n* = 1 each). Three had a ‘typical’ autistic regression phenotype (cases 1, 3, 6) and three had features reminiscent of paediatric acute‐onset neuropsychiatric syndrome^7^ (PANS; cases 1, 2, 6). Three patients had ongoing infection‐provoked exacerbations.

The patients were followed for an average of 8 years (range 1–16 years) and have ongoing neurodevelopmental disorders (Table 2). Four have significant immune disorders (cases 2, 3, 4, 6); two had documented immune deficiency (IgA deficiency and Ig deficiency; cases 3, 4); a further two had recurrent severe infection history (cases 2, 6); two had autoimmune diseases (cases 3, 6), and one has hidradenitis suppurativa on anti‐IL23 therapy (case 6). Four had other organ disease (congenital heart disease in two).

Seven children benefited from conventional psychiatric treatments (Table 2), six received immune‐modulating treatments (three intravenous immunoglobulin, four antibiotics, one rituximab), and one patient was treated with the ketogenic diet.^6^ According to the clinician and parents, there was significant and justifiable benefit of intravenous immunoglobulin (cases 1, 5, 7) and antibiotics (cases, 1, 2, 3, 6).

### Case reports

Case histories are profiled with two different dominant phenotypes (PANS like, autistic regression). The other six cases are presented in supplementary material (Appendix S1).

### Case 3 (PANS like, Koolen de Vries, *KANSL1* )

This male was born at 36 weeks, weighing 2.1 kg. Dysmorphism was noted at birth, followed by global developmental delay, hypotonia, epilepsy, and a tethered spinal cord. He also had multiorgan problems, including IgA immune deficiency, Hashimoto thyroiditis, eosinophilic and fungal oesophagitis, and growth hormone deficiency. At age 6 years 6 months, the day after a short hospital admission for upper respiratory tract infection induced croup, he experienced abrupt changes in function and personality, with social withdrawal, reduced interaction, new onset separation anxiety, new sensory symptoms (sound and touch), and repetitive, ritualistic, and compulsive behaviours such as flapping, clapping, grunting, pacing, and using his face to touch the ground. Over the following year, these symptoms progressively deteriorated, requiring fluoxetine, risperidone, aripiprazole, methylphenidate, pregabalin, and lithium. Erythromycin was trialled (3 years after PANS onset) and produced significant improvements in prosocial intent, anxiety, attention, and concentration, back to his baseline before regression. By contrast, his repetitive behaviours and sensory sensitivities remained unchanged. He continued erythromycin 250 mg twice per day for 2 years 6 months alongside his conventional psychiatric medication.

### Case 6 (autistic regression and PANS like, Rahman syndrome, *HIST1H1E* )

This male was born at term, with an older brother who has autism spectrum disorder level 1. He was considered typically developing at 1 year, with 25 single words including man, dad, ball, and dog, and purposeful eye contact and social interaction. The day after his measles, mumps, and rubella vaccination at age 12 months, he developed a fever and stopped speaking, lost eye contact, and began repetitive and stereotypical movements including arm slapping. He was diagnosed with autism spectrum disorder level 3 at 18 months. He developed compulsive behaviours, and had sensory issues to sound, light, and food textures. However, he was calm, affectionate, and gentle, and able to be directed. From early childhood, he had recurrent infections with urinary tract infections, ear infections, tonsilitis, recurrent skin boils (four per year), and significant vulnerability to croup. In addition, his compulsive behaviour became a more challenging priority. At age 11 years, he had a mycoplasma pneumonia requiring admission to hospital. One week later, he suffered an abrupt deterioration in his behaviour, with anxiety and OCD behaviours, including refusing to enter his home, resulting in the family having to live in a caravan outside the house (ongoing). He developed obsessions about putting rubbish in bins and would put imaginary rubbish in his parent's hands. He developed repetitive tic‐like behaviours including hitting his stomach and chest. His general behaviour deteriorated with increasing anger, aggression, and rage, and his sleep became fragmented, often not sleeping for 36 hours. He developed type 1 diabetes, had recurrent diarrhoea and skin boils, and hidradenitis suppurativa requiring immune therapy. Efforts to treat the symptoms with conventional treatments failed because of his refusal to take the medicine. A trial of azithromycin 500 mg once per day for 1 month improved sleep and aggression, with reduced OCD, and more flexibility. Unfortunately, after 1 month, he refused to take the medicine any longer, and the poor sleep, OCD, outbursts, and challenging behaviours returned.

### Cell type RNA sequencing

**FIGURE 2:**
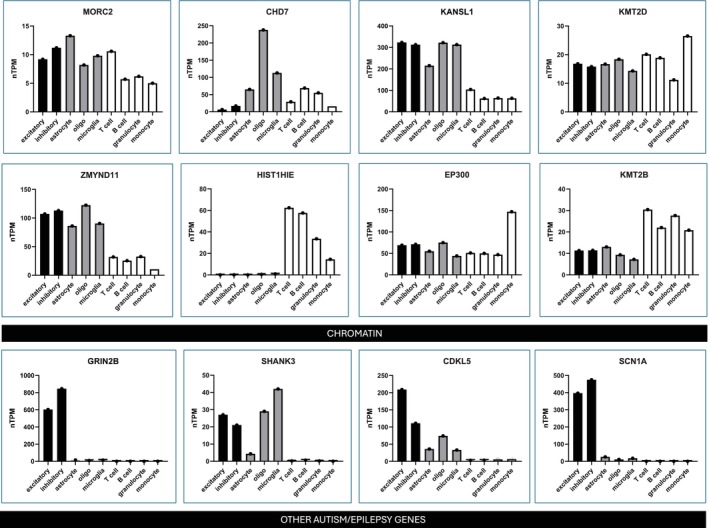
Normalized transcripts per million values from proteinatlas.org using single‐cell ribonucleic acid (RNA) sequencing data sets are presented for each gene, for neuronal cells (excitatory and inhibitory) in black, glial cells (astrocyte, oligodendrocyte, and microglia) in grey, and peripheral leukocytes (T cell, B cell, granulocyte, and monocyte) in white. The chromatin genes (*MORC2, CHD7, KANSL1, KMT2D, ZMYND11, HIST1H1E, EP300, KMT2B*) reveal generally equal expression across neuronal, glial, and peripheral blood cell types, whereas selected non‐chromatin neurodevelopmental genes (*GRIN2B, SHANK3, CDKL5, SCN1A*) reveal RNA expression predominantly in neuronal and glial cells.

The normalized transcripts per million for the nine cell types was plotted for the eight chromatin genes. For comparison, we present four selected non‐chromatin genes that cause neurodevelopmental syndromes (two of the most common causes of genetic autism [*GRIN2B*, *SHANK3*] and two of the most common causes of genetic epilepsy [*SCN1A*, *CDKL5*]) (Figure 2). The chromatin genes generally showed broad RNA expression across all cell types and were expressed in peripheral leukocytes. By contrast, in the non‐chromatin genes (*GRIN2B*, *SHANK3*, *CDKL5*, *SCN1A*), RNA expression was high in neurons and sometimes glia, but generally low or undetectable in peripheral leukocytes.

## DISCUSSION

Neurodevelopmental disorders such as autism spectrum disorder, Tourette syndrome, and attention‐deficit/hyperactivity disorder affect 10% of all children, and have a significant impact on individuals, families, society, and health care systems.^8^ The yield of genetic testing in people with autism spectrum disorder is higher when associated with dysmorphism, intellectual disability, or epilepsy (30–50%), but lower in the absence of these associated features (often <10%).^9^, ^10^

These eight cases all have in common a highly penetrant, rare de novo pathogenic DNA variant in genes associated with chromatin formation. However, there was a high rate of neurodevelopmental disorders in first‐degree family members, suggesting the possibility of other vulnerability genes. There was also a high rate of autoimmune or inflammatory disorders, particularly in the mothers, which raises the possibility of other immunogenetic factors or ‘maternal immune activation’ during pregnancy which could play an additive role in the expression of the unusual phenotype in these patients.^11^, ^12^ It is possible that these cumulative factors have resulted in these regression syndromes (rare and common genomic variants plus environmental factors).

The commonality in these patients was the presence of infection‐provoked neuroregression and/or abrupt‐onset neuropsychiatric disease, which was typically incapacitating, dangerous (facial haematomas, status epilepticus), or impairing. We believe these infection‐provoked deteriorations represent an example of gene–environment interactions—specifically the impact of infection or vaccination (environmental immune stimuli) interacting with a dysregulated immune system and neurological system.^11^ The RNA sequencing demonstrates that RNA of these chromatin genes is diffusely expressed across immune and brain cells. We hypothesize that immune cells with aberrant gene expression will react abnormally to immune stimuli, and the corresponding crosstalk with neurons and glia, which also have aberrant gene expression, will result in deteriorations in neurodevelopmental trajectories. Neurodevelopment is an active process of evolving synaptic connectivity, and synaptic pruning is an immune process performed by microglia via complement mediated phagocytosis.^13^ Some of these patients had clinical phenotypes reminiscent of autistic regression and PANS although the clinical phenotypes were heterogeneous also including motor, executive, cognitive, and seizure features.

Although one patient (case 5) had a definite encephalitis as the initiating provoking factor, there was no evidence of encephalitis during or after the regression phases using conventional testing, suggesting that the immunopathogenic process is different to encephalitis.

Although conventional medications did help challenging behaviours, immune therapies were also useful in improving the developmental trajectories in some patients. Some of the observations were serendipitous, such as families noting that the neurological symptoms improved when antibiotics were used to treat infections (azithromycin). It is acknowledged that our therapeutic observations are vulnerable to observer bias, and subsequent observations are more likely to demonstrate ‘regression to the mean’. Further studies should explore the mechanisms of action of these therapies using transcriptomic, proteomic, or functional assays.

The small sample size of patients investigated specifically for infection‐provoked episodes of neuroregression and abrupt‐onset neuropsychiatric disorders is a limitation of this study. Further studies auditing presence of these manifestations in larger cohorts of children with similar genetic variants or other chromatin gene‐associated disorders may support the findings and identify the magnitude of polygenic and/or environmental contributions. Parental observation and clinician perception was that the regressive episodes in these children were often triggered by infections; however, we acknowledge that infectious disease is common in children, which renders such infection‐associated clinical observations vulnerable to false association.

In summary, Mendelian disorders of chromatin genes result in combined immune dysregulation and neurodevelopmental syndromes. These cases show the role of chromatin dysregulation in immune‐brain interactions, and provide a model of poorly understood clinical syndromes such as autistic regression and PANS.

## Supporting information


**Appendix S1:** Case reports.

## Data Availability

The data that support the findings of this study are available on request from the corresponding author. The data are not publicly available due to privacy or ethical restrictions.
