# Supplementary material for: Pathogenic variants in chromatin‐related genes: Linking immune dysregulation to neuroregression and acute neuropsychiatric disorders
Source: Dev Med Child Neurol. 2025 Feb 22;67(8):1095–102. doi: 10.1111/dmcn.16276 (PMC12237219; doi:10.1111/dmcn.16276)
Supplement: Supplementary file 1 — Appendix S1: Case reports. [file DMCN-67-1095-s001.docx]

**Appendix S1**

**Case Reports - CLINICAL COURSE**

**Case 1**

This girl had a normal early development until 4.9 years of age. At that time described as a very sociable, cheerful, inquisitive little girl, had extensive vocabulary, who enjoyed every day social interactions, social play, maintained friendships, and was able to speak fluently in two languages, copy letters and words and demonstrated confidence in learning.

Age 4.9 she had gastroenteritis, two episodes of tonsillitis and one URTI over 2 months and the final infection merged into ongoing vomiting, headache and photophobia. Two weeks of the last infection, she was incontinent of urine and stool, had hallucinations, her personality changed and she appeared fearful, had separation anxiety, loss of eye contact, had eating restriction and went back into nappies. Over the following year, her eating improved but her behaviour worsened, with agitation, inattention, aggression, oppositional behaviour, hallucinations and smearing faeces. She had two seizures which requiring ASM. She was unable to follow-rules, had unsustained focus, would fight and flight from school, and she could no longer read or write any words. Her separation anxiety resulted in her mother having to accompany her to the toilet and do all activities including dressing and feeding. Every infection, however mild, resulted in deterioration in emotional control, tantrums, worsening coordination, and worsening hallucinations. She could only attend one day of school per week.

MRI and CSF was negative including autoantibodies. Conventional treatment produced mild calming benefits. 2 years after onset, given the ongoing infection provoked relapsing course, intravenous immunoglobulin was started, and has produced benefits including making developmental progress for the first time in 2 years, reduction in separation anxiety and hallucinations. On the IVIG, she has started self-feeding with cutlery for the first time in 2 years, she has been able to go on trips, her socialisation and interest in other children as improved, her play has become normal, and she has started writing and copying again and making academic progress for the first time in 2 years. However, the clinical course is not smooth- infections continue to result in some deteriorations and amoxicillin/clavulanic acid has reduced the severity of these infection provoked deteriorations. Currently, she attends school every day, is learning, can speak in full and complex sentences, is more engaged, more empathetic and emotionally controlled.

**Case 2**

This girl’s cardiac abnormalities were detected during pregnancy and cardio-respiratory problems required a 3 month neonatal inpatient stay. Sensorineural hearing loss plus choanal atresia resulted in a diagnosis of CHARGE syndrome. Three cardiac surgeries, three corrective surgeries for the choanal atresia, plus insertion of a cochlear implant were required. She required warfarin anticoagulation due to aortic and mitral valve replacements. There was global developmental delay from the first year of life, she walked independently from age 5 years, she is non-verbal but uses sign language. She had recurrent respiratory infections requiring intravenous antibiotics every 2-3 months between age 1-4 years. In addition, ear infections occurred 2-3 times per year plus urinary tract infections 1-2 times per year, an episode of pseudomonas sepsis, cochlea implant infection, all requiring antibiotics. After each infection she would cease eating, and after one infection her eating regressed to only eating baby formula and she only resumed eating solids after a year.

From age 5 she has had increasing compulsive behaviour including touching her mother’s hand or the car. If someone dropped something on the floor, she would become hysterical. From age 5 she also developed motor tics. She was diagnosed with autism age 10 years. From 8 years of age, she developed increasing self-injurious behaviour, involving punching herself in the face, and her anticoagulation caused dangerous haematomas and facial bleeding. In conjunction, OCD behaviours involving her parents tapping her hands 100 times were incapacitating. Her mother noted that nurofen improved the self-injurious behaviours, and after an episode of mycoplasma pneumonia treated with azithromycin, there was complete cessation of the self-injurious behaviour. Three further courses of azithromycin over one month produced a remission of her self-injurious behaviour for one year, but after a further cardiac surgery, the behaviours returned. A trial of azithromycin 375mg four times a week was tolerated and resulted in substantially reduced self-injury, agitation, compulsive tapping behaviours and a return of her happy and compliant behaviour occurred. Her parents also note that her gut health and eating behaviours appear to be associated with her behavioural control. She remains on azithromycin 375mg four times a week, and her ECG QT is normal.

**Case 4**

This male patient required cardiac repair for coarctation repair after birth and subsequent aortic value replacement. He had visuospatial processing problems but otherwise had normal cognition. He had immune deficiency, recurrent ear infections, visual impairment with coloboma, hearing impairment, hip dysplasia, growth hormone deficiency, and incidental von Willebrand disease. He was diagnosed with Kabuki syndrome at age 4 years. From 2.5 years of age he had recurrent urine tract infections and ear infections. From 2.5 years of age, it was noted that every infection (ear, upper respiratory tract infection) would provoke a neuro-regression of ‘brain fog’ with inattention, memory loss of learned information and reduced interaction. These regression episodes would last 1-2 months, then he would return to his previous baseline, but these relapsing remitting events, occurring 2 times per year on average, resulted in some cognitive stagnation. At 12 years of age, a ketogenic diet was started as a disease modifying therapy of his epigenetic disorder, due to the known HDAC inhibitor effects of butyrate. Over the last 4 years on the ketogenic diet, his infection provoked brain fog events have ceased, his recurrent ear infections have ceased, and his baseline hearing function improved, and his cognitive and learning function has improved, enabling him to stay in his mainstream school. He has also benefited from methylphenidate for his ADHD.

**Case 5**

This male patient had some mild speech delay and delayed social development but had not needed to seek medical assessment before the episode of encephalitis aged 5 years. After an episode of gastroenteritis, he presented with acute encephalopathy and seizures, and MRI showed widespread inflammatory changes in the cortical grey matter and deep grey matter, with a CSF pleocytosis (17 polymorphs, 38 monocytes) and elevated CSF neopterin of 178 nmol/l (normal<30), but autoantibodies were negative. He was treated with 3 days of intravenous methylprednisolone and 2g/kg of intravenous immunoglobulin and made a partial recovery over the following 2 months. However, over the following 2 years after encephalitis, he developed narcolepsy, focal epilepsy, cognitive decline, tics, and increasing challenging behaviour and was diagnosed with ASD. Repeat imaging and CSF investigations were normal. Given the deteriorating neuropsychiatric syndrome, intravenous immunoglobulin was started and improved control of his epilepsy, tics, cognition and behavioural syndrome. He became immune therapy dependent and needed 2g/kg of IVIG 3 weekly and rituximab re-dosing every 4 months, in addition to multiple conventional psychiatric agents. Other medications trialled which were not useful included mycophenolate mofetil, ruxolitinib and baricitinib. Attempts to reduce the dose or increase the duration of IVIG or rituximab resulted in status epilepticus, deterioration in cognition, incontinence, visual hallucinations, sleep fragmentation, dysarthria, tics and behavioural decline (aggression and self-injury), particularly in the week that IVIG was due, or when B cells repopulated. The working hypothesis was of a post-encephalitic chronic immune responsive neuroimmune disorder.

**Case 7**

This boy’s mother was diagnosed with a somatic JAK2 variant in the context of a haematological disorder and history of thyroid cancer. He had speech delay (short sentences, echolalia at 7 years of age) and had been assessed for ASD but did not meet criteria. He was reported to be fidgety in class with poor concentration. His motor development was age appropriate. Age 7 years, he presented after 3-4 days of an upper respiratory illness with fever with sudden behavioural change (irritable, crying, clingy) and was unable to walk. He was investigated and found to have an acellular CSF but his CT scan showed calcifications involving bilateral globus pallidus, grey-white matter junction of bilateral frontal and left temporal lobe and choreoid plexus, corresponding to areas of increased susceptibility on MRI. He was treated with a pulse of 20 mg/m2/day of dexamethasone for 3 days and 2 g/kg IVIg. He resumed walking at discharge 6 days after treatment initiation. He received a second pulse 4 weeks after the first with a 3 day course of dexamethasone 20mg/m2/day and IVIG 1g/kg. There were no further episodes of regression over the following year. His school assessment noted him to be making progress but behind in most areas compared to peers. He has ongoing mood lability and some anxiety. He was also noted to have bilateral upper limb hyperreflexia.

**Case 8**

This boy was born at 34+2 weeks and had symmetric IUGR. He had some delay in walking (15 months). Prior to the regression, he was using 5-6 words and short sentences, responsive to his name and following commands, and was able to walk and run. After the febrile URTI at 2 years of age, he lost speech and when seen at 3 years of age, he was only babbling. He used to be able to hold a spoon, which also stopped after the illness. After the illness at 2 years of age, he stopped responding to his name and was inconsistent in following commands. His gait also worsened, and he developed toe walking with frequent falls. He was assessed to have normal hearing. He was investigated 8 months after this period of regression with a lumbar puncture, normal neuroimaging and genetic testing. At 6 years of age, he has hyperactivity, sleep difficulty and persisting learning difficulties and growth parameters <3^rd^ centile. He has formally been diagnosed with autistic spectrum disorder.
